# Potential Protein Target to Therapeutically Restore Positive Mood States in Depression

**DOI:** 10.1523/ENEURO.0105-19.2019

**Published:** 2019-03-28

**Authors:** Rosalind S.E. Carney

## Abstract

**Highlighted Research Paper:** Disorders of the Nervous System Highlighted Research Paper: p11 in Cholinergic Interneurons of the Nucleus Accumbens Is Essential for Dopamine Responses to Rewarding Stimuli, by Y. Hanada, Y. Kawahara, Y. N. Ohnishi, T. Shuto, M. Kuroiwa, N. Sotogaku, P. Greengard, Y. Sagi, and A. Nishi.

Major depression is one of the most common mental health disorders; meta-analysis of 83 cross-sectional studies found a 27% pooled prevalence of depression or depression-like symptoms among outpatients (Wang et al., 2017). Symptoms of depression include mood states of increased negative affect such as depressed mood, suicidality, fatigue, guilt, reduced focus, and feelings of worthlessness (American Psychiatric Association, 2013). Antidepressant treatments that target serotonergic signaling help alleviate some of these symptoms. However, current treatments do not adequately mitigate symptoms of decreased positive affect in which individuals experience less interest in social and physical interactions. This loss of interest in normally pleasurable events, termed anhedonia, is associated with a reduction in dopaminergic neurotransmission (Der-Avakian and Markou, 2012; Russo and Nestler, 2013). In their *eNeuro* publication, Hanada et al. (2018) found that the p11 (S100A10) protein, a member of the S100 EF-hand family, functions in reward-mediated dopamine release, and therefore therapeutic strategies that increase the function of p11 may have an impact on antidepressant efficacy in alleviating anhedonia.

The mesolimbic reward circuit is mediated by dopaminergic input from the ventral tegmental area to the nucleus accumbens (NAc). Within the NAc, dopamine release is stimulated by the release of acetylcholine (ACh) from cholinergic interneurons (CINs), which express high levels of p11. Postmortem tissue from humans with depression reveals reduced p11 expression in the NAc (Svenningsson et al., 2006; Alexander et al., 2010). Restoration of p11 expression locally in the NAc of *p11* knock-out (KO) mice reverses depression-like behaviors (Alexander et al., 2010). When *p11* is specifically deleted in choline acetyltransferase (ChAT)-expressing cells (*ChAT-p11* cKO) in the NAc, mice exhibit depression-like behaviors (Warner-Schmidt et al., 2012). These prior studies confirmed that p11 in CINs of the NAc regulates depression-like behaviors, but a direct link between p11 and dopaminergic transmission had not yet been established. To investigate whether p11 could directly impact dopaminergic neurotransmission, Hanada et al. (2018) placed microdialysis probes in the NAc of wild-type (WT), *p11* KO, and *ChAT-p11* cKO mice. Microdialysis enabled real-time measurements of changes in extracellular neurotransmitter levels *in vivo* upon exposure to one of three reward stimuli.

In WT mice, exposure to cocaine, palatable food, or a female mouse increased extracellular dopamine (DA) levels in the NAc. In *p11* KO mice, the DA response was largely attenuated in response to cocaine infusion and was abolished following exposure to palatable food or a female mouse. These results indicate that p11 selectively regulates DA-mediated reward responses in the NAc. The authors then assessed the effects of ACh receptor activation in *p11* KO mice using nicotinic ACh receptor (nAChR) and muscarinic ACh receptor (mAChR) agonists. When the nAChR agonist, nicotine, or a non-selective mAChR agonist, oxotremorine, was co-infused into the NAc with cocaine, extracellular DA levels in *p11* KO mice were increased similarly to those in WT mice. These experiments indicate that when ACh receptors presumably located at dopaminergic terminals are stimulated, the DA response to cocaine is restored in *p11* KO mice. Therefore, endogenous p11 functions to stimulate ACh release and regulates the ACh-mediated DA response to rewarding stimuli.

To confirm that p11 in NAc CINs mediates the DA response to rewarding stimuli, Hanada et al. (2018) repeated the microdialysis experiments in *ChAT-p11* cKO mice. Loss of p11 function in ChAT-expressing cells resulted in attenuated DA responses to cocaine infusion, palatable food, or female exposure in the NAc (Fig. 1). These attenuated DA responses were restored by overexpression of p11 in NAc CINs of *ChAT-p11* cKO mice. Chemogenetic alteration of CIN activity in *ChAT-p11* cKO mice also supports the concept that p11 function in CINs is a critical regulator of the DA response to rewarding stimuli in the NAc. Microdialysis measurement of extracellular ACh levels after cocaine infusion into the NAc of *ChAT-p11* cKO mice confirmed the concept that p11 functions endogenously in CINs to regulate ACh release, which in turn enhances DA release in the NAc.

**Figure 1.**
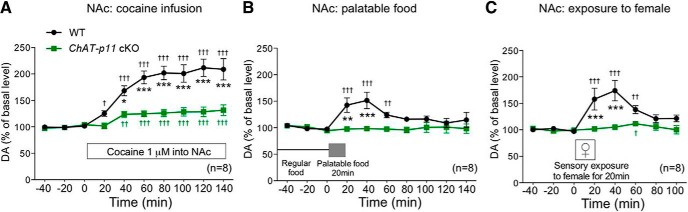
DA response to rewarding stimuli in the NAc of *ChAT-p11* cKO mice. Extracellular levels of DA in the NAc of WT and *ChAT-p11* cKO mice following cocaine infusion into the NAc (***A***), exposure to palatable food (***B***), and exposure to female mice (***C***). The basal values for each group were obtained as the average of three stable baseline samples, and all values are calculated as a percentage of the basal values within the same group (100%). Data represent mean ± SEM. *p < 0.05, **p < 0.01, ***p < 0.001 versus WT mice; two-way ANOVA and Bonferroni multiple comparison: †*p* < 0.05, ††*p* < 0.01, †††*p* < 0.001 versus the basal levels of DA in the same group. The number of mice is indicated in parentheses (adapted from Hanada et al., 2018, their Fig. 3).

This *eNeuro* publication is an important advance in the field because the findings implicate loss of p11 function as a critical factor that results in the attenuated ACh release and impaired dopaminergic transmission that underlies anhedonia (Fig. 2). As there is an association between anhedonia and suicidality, substance abuse, and schizophrenia (Craske et al., 2016; Loas et al., 2018), it is imperative to develop therapeutic treatments that maintain positive affect in depression. Positive and negative affects are not extreme mood states on the same mood spectrum; rather they represent two independent dimensions (Watson and Clark, 1997). Therefore, it will hopefully be possible to maintain or increase positive affect and simultaneously decrease negative affect in depressed individuals. However, as p11 potentiates serotonergic transmission (Svenningsson et al., 2006; Warner-Schmidt et al., 2009), it is not known how difficult it would be to achieve dual-affect treatment(s). But for now, this *eNeuro* publication opens up the potential for the development of antidepressant treatments that can reduce symptoms of anhedonia in individuals with depression.

**Figure 2.**
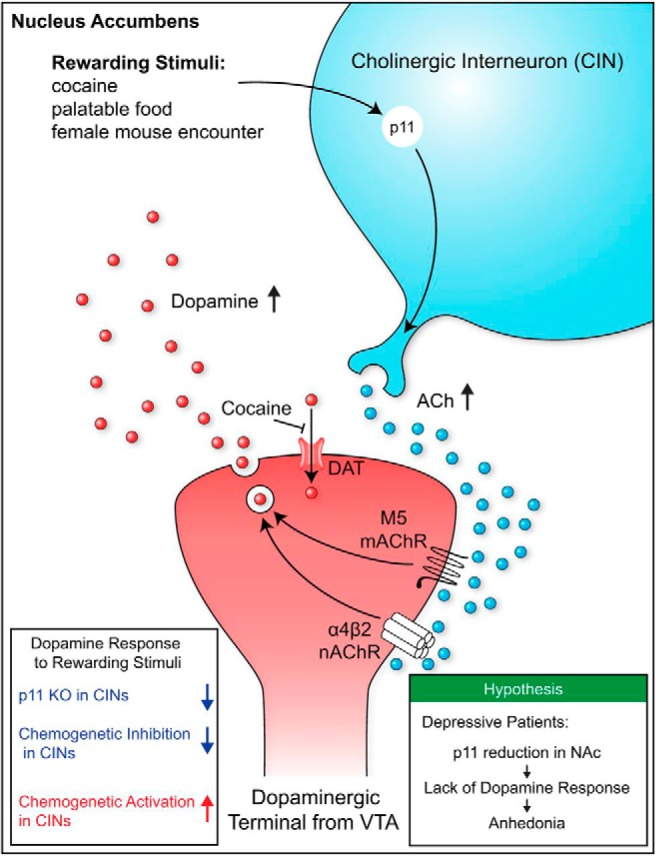
Schematic illustration of the role of p11 in the dopamine response to rewarding stimuli (visual abstract from Hanada et al., 2018).
